# Syphilinum, in Kidney Disease, &c.

**Published:** 1881-03

**Authors:** Frances Burritt

**Affiliations:** New York


### A CASE OF KIDNEY AND BLADDER IRRITATION CURED BY SYPHILINUM.

BY FRANCES BURRITT, M.D., NEW YORK.

C. H. B., aged 42, dark complexion.

July 11th, 1872. Called on me and complained of great pain in the back, in the region of the kidneys, worse after urination. Micturition difficult and very slow; no pain, but a want of power, so that he has to strain to pass it.

Redness and rawness with terrible itching between the toes, worse at night. Eighteen years before had been treated for syphilis allopathically, since which time, though not before, had been constantly troubled with the above symptoms.

Gave *Syphilinum* 1 M; in twelve hours urinated freely.

July 12th. Was taken with great pain in the head; the whole body was extremely cold, looked blue, wanted to be covered with blankets, yet could not get warm; no appetite, but sleeping almost all the time, could scarcely be aroused. This continued for five days, and I was then sent for.

July 17th. Above condition still continued; also discovered that an eruption was making its appearance over the whole body; the eruption was not elevated, but could be distinctly felt by passing the hand over the skin.

Gave *Syph.* 1 M. Head almost immediately relieved; slight perspiration over the whole body, eruption rapidly coming to the surface, at the same time a disagreeable odor began to be developed; eruption was reddish brown, resembling smallpox pustules; the body was literally covered, with the exception of the scrotum and penis.

A large bubo had also made its appearance in the right groin, but as there had been no pain, the patient had not noticed it before.

July 29th. *Syph.* 1 M. Bubo opened, and discharged very freely.

Eruption much worse, completely covering the inside of the mouth and throat, making it difficult to swallow even liquids; eyes also covered, making him completely blind. Intolerable smell from the body.

Aug. 15th. Consulted Dr. Swan, who gave *Nit.-ac.*; no perceptible effect.

Aug. 30th. Again consulted Dr. S., who gave *Merc.* 40 M. No better, but eruption generated a great quantity of pus, with intolerable itching, yet could not scratch, as it was extremely sore.

Bone-pains in the knees and feet.

He then began slowly to mend, but was not able to attend to business till November. He has not had any return of the former symptoms since the first relief; the feet are well, and the urinary organs completely cured. Has lost almost all his hair.

In October his son (aged nine years) complained of a gathering in his left ear, which discharged a great quantity of pus; left side of nose, inside and out, very sore; lips and chin also. Sores itching and scabbing over.

Soon after his daughter (aged thirteen years) commenced breaking out with a similar eruption on the left side of the nose, lips, and chin.

Thinking that the children had been poisoned by the atmosphere while the father was sick (as they had never had any eruption before), gave to each of them one dose of *Syph.* 1 M.

Sores much improved in twelve hours, many drying up, and the scabs falling off, leaving the skin beneath a dull reddish copper color; improvement continued very rapid, being nearly all cured in a week or ten days.

